# Investigation of the underling mechanism of ketamine for antidepressant effects in treatment-refractory affective disorders via molecular profile analysis

**DOI:** 10.3892/etm.2019.7633

**Published:** 2019-05-30

**Authors:** Jun Qiao, Yuan Sun, Jinfang Wu, Li Wang

**Affiliations:** 1 Department of Psychiatry, The First Hospital of Hebei Medical University, Shijiazhuang, Hebei 050000, P.R. China; 2 Department of Anesthesiology, The First Hospital of Hebei Medical University, Shijiazhuang, Hebei 050000, P.R. China; 3 Department of Anesthesia Surgery, The First Hospital of Hebei Medical University, Shijiazhuang, Hebei 050000, P.R. China

**Keywords:** ketamine, antidepressant effect, molecular profile

## Abstract

Ketamine elicits a rapid antidepressant effect in treatment-refractory affective disorders. The aim of the present study was to elucidate the underlying mechanism of this effect and to identify potential targets of ketamine for antidepressant effects. GSE73798 and GSE73799 datasets were downloaded from the Gene Expression Omnibus database. Differentially expressed genes (DEGs) were identified in hippocampus or striatum samples treated with ketamine, phencyclidyne or memantine compared with a saline or normal group at 1, 2, 4 and 8 h. The overlapping DEGs were the DEGs in both hippocampus and striatum samples. Kyoto Encyclopedia of Genes and Genomes and BioCyc databases were used to perform functional annotation and pathway analyses. Protein-protein interactions (PPIs) were predicted using Search Tool for the Retrieval of Interacting Genes/Proteins version 9.1 for the DEGs in the striatum samples treated with ketamine, phencyclidine or memantine compared with normal samples. Reverse transcription-quantitative polymerase chain reaction was performed to determine mRNA levels. Perilipin 4 *(Plin4)*, serum/glucocorticoid regulated kinase 1 *(Sgk1)*, kruppel like factor 2 *(Klf2)* and DDB1 and CUL4 associated factor 12 like 1 *(Dcaf12l1)* were the overlapping DEGs in the striatum samples treated with the three drugs at different time points. The mRNA expression levels of *Plin4, Sgk1* and *Klf2* were significantly higher (P<0.05), and the mRNA expression level of *Dcaf12l1* was significantly lower in the striatum samples of the ketamine-treated group compared with the control group in an *in vivo* experiment. Both *Sgk1* and *Klf2* were enriched in the ‘forkhead box O (FoxO) signaling pathway’, and *Sgk1* was additionally enriched in the ‘mechanistic target of rapamycin kinase (mTOR) signaling pathway’. PPI networks of DEGs in the striatum samples treated with ketamine, phencyclidine and memantine compared with normal samples were constructed, and *Klf2* was involved in more pairs and was therefore a gene hub in the three networks. The four genes, *Plin4, Sgk1, Klf2* and *Dcaf12l1*, were differentially expressed in all of the groups that treated with the three drugs and their expression levels were verified in *in vivo* experiments. The FoxO and mTOR signaling pathways may be involved in the underlying mechanism of the antidepressant effects of ketamine, and *Plin4, Sgk1, Klf2* and *Dcaf12l1* may be potential biomarkers for depression in N-methyl-D-aspartic acid receptor antagonist treatment.

## Introduction

Depression is a state of low mood and aversion to activity that can affect a person's thoughts, behavior, feelings and sense of well-being (1). It has been predicted that by 2030, depression will account for the highest level of disability among all physical and mental disorders worldwide (2). People with depression may feel sad, anxious, empty, hopeless, helpless, dejected or worthless (3). Certain life events or changes may contribute to depression, including childbirth, menopause, financial difficulties, stress, social isolation or relationship difficulties (4). Adolescents are especially prone to experiencing depression following social rejection, peer pressure and bullying (5). In addition, several diseases (including hypothyroidism, multiple sclerosis, Parkinson's disease and chronic pain), and drugs (including heroin, intoxication, hallucinogens and inhalants) can cause or exacerbate depression (6,7). Depression is also a symptom of treatment-refractory affective disorders (8). As indicated in the aforementioned studies, depression may be a temporary reaction to life events, a symptom of a medical condition, or a side effect of certain drugs or medical treatments.

N-methyl-D-aspartic acid (NMDA) receptor antagonists are a class of anesthetics that inhibit the action of NMDA receptors. Ketamine, phencyclidine and memantine are three common drugs belonging to the class of NMDA receptor antagonists (9,10). They act primarily on the nervous system, with mild stimulant effects at subanesthetic doses, and effects of dissociation and hallucinations at higher doses (11). Ketamine is the primary anesthetic for burn victims and emergency patients with unknown medical history due to its moderate inhibition of respiration and circulation (12,13). Certain studies have indicated that NMDA antagonists also exert antidepressant effects, particularly ketamine (14–16). However, the underlying molecular mechanism of this remains unclear. Therefore, the aim of the current study was to explore the underlying mechanism, and identify potential targets of ketamine for antidepressant effects based on molecular profile analysis.

## Materials and methods

### 

#### Expression profiles

mRNA expression profiles GSE73798 and GSE73799 were downloaded from the publicly available Gene Expression Omnibus (GEO) database (www.ncbi.nlm.nih.gov/geo). The GSE73798 profile contained data derived from 60 mouse hippocampus samples treated with ketamine, phencyclidyne, memantine and physiological saline or no treatment assessed at four time points (1, 2, 4 and 8 h after treatment). The GSE73799 profile included data derived from 60 mouse striatum samples that underwent the same treatments and assessments. Samples had been evaluated with the Illumina MouseWG-6 version 2.0 Expression BeadChip platform (Illumina, Inc., San Diego, CA, USA). A preliminary study using mice failed to establish and investigate the model of depression, and, therefore, a rat model was used in the present study to confirm differential gene expression.

#### Data processing and differentially expressed gene (DEG) analysis

Raw data were obtained and normalized with preprocessCore package (version 1.32.0; www.bioconductor.org/packages/3.2/bioc/html/preprocessCore.html), and probe symbols were converted to gene symbols. Subsequently, DEGs were identified in hippocampus or striatum samples individually treated with the three drugs, saline or no treatment and assessed at 1, 2, 4 and 8 h, respectively. Each sample was analyzed three times. P<0.05 and |log(fold-change)|>0.05 were used as the threshold criteria. A total of 48 sets of DEGs were obtained. Overlapping DEGs in the 24 sets were screened out for subsequent evaluation.

#### Functional annotation and pathway analysis

Kyoto Encyclopedia of Genes and Genomes (KEGG release 82; www.genome.jp/kegg) and BioCyc (version 21.0; biocyc.org) databases were used to perform functional annotation and pathway analysis for the overlapping DEGs. Gene ontology (GO) terms and pathway terms were selected at P<0.05.

#### Construction of the protein-protein interaction (PPI) network

From the results of screening DEGs, the authors found that the three drugs had a greater effect on gene expression in striatum samples, therefore striatum samples were selected for further study. The search tool for the retrieval of interacting genes/proteins (STRING; version 9.1; string-db.org) is a biological database and web resource of known and predicted PPIs. In the current study, PPIs with a confidence score >0.4 were selected using STRING for the DEGs in the striatum samples treated with ketamine, phencyclidyne, memantine compared with normal samples. The PPI network was constructed using Cytoscape software (version 3.5.1; www.cytoscape.org/download.php).

#### Verification of associated genes

A total of 10 male Sprague-Dawley rats (age, 12 weeks; weight, 300–350 g) were purchased from Beijing Vital River Laboratory Animal Technology Co., Ltd. (Beijing, China). They were fed in specific-pathogen free (SPF) facilities under standard conditions and had access to food and water. The rats were housed at 18–29°C with a 12 h light/dark cycle and a relative humidity of 40–70%; the rats had access to food and water *ad libitum*. A depression model was constructed using the chronic unpredictable mild stress method (17). The rats were randomly divided into a ketamine group and a control group, with 5 rats in each group. Ketamine (25 mg/kg; BOC Science Co., Ltd., Shirley, NY, USA) was injected intraperitoneally in the ketamine group, while a similar volume of sterile saline was injected intraperitoneally in the control group (18). At 8 h after drug administration, the rats were sacrificed via cervical vertebrae dislocation after anesthesia with pentobarbital sodium (45 mg/kg; intraperitoneal) and striatum samples were collected. All rat experiments were approved by the Animal Use and Care Committee of the First Hospital of Hebei Medical University (Shijiazhuang, China). Reverse transcription-quantitative polymerase chain reaction (RT-qPCR) (19) was performed to determine the mRNA expression levels of perilipin 4 *(Plin4)*, serum/glucocorticoid regulated kinase 1 *(Sgk1)*, kruppel like factor 2 *(Klf2)*, and DDB1 and CUL4 associated factor 12 like 1 *(Dcaf12l1)* with the ABI Am1005 AgPath-ID™ One-Step RT-PCR kit (Invitrogen; Thermo Fisher Scientific, Inc., Waltham, MA, USA) according to the manufacturer's protocol. The 2^−ΔΔCt^ method (20) was used to quantify the results. The following primer sequences were used: *Plin4* 5′-GGGACAAGAACATGGGAAGC-3′ (forward) and 5′-CCTTGACAAGACCTTTGGCC-3′ (reverse); *Sgk1* 5′-GAAGCTTGCCAACAACTCCT-3′ (forward) and 5′-CGTGGGGATTTGAGGATGGA-3′ (reverse); *Klf2* 5′-CTATCTTGCCGTCCTTTGCC-3′ (forward) and 5′-GGCTCCGGGTAGTAGAACG-3′ (reverse); *Dcaf12l1* 5′-CAGCAGCAAACAGGTAGCAG-3′ (forward) and 5′-CCTACCTCCCGAACCTTCAG-3′ (reverse). β-actin was as used as an internal reference, and the primer sequences were 5′-CTACAATGAGCTGCGTGTGG-3′ (forward) and 5′-AGGCATACAGGGACAACACA-3′ (reverse).

#### Statistical analysis

SPSS software (version 17.0; SPSS, Inc., Chicago, IL, USA) was used for all statistical analyses and data were expressed as the mean ± standard error of the mean. Student's t-test was used to compare groups. P<0.05 was considered to indicate a statistically significant difference.

## Results

### 

#### DEGs

A total of 48 sets of DEGs were identified in hippocampus or striatum samples individually treated with the ketamine, phencyclidine or memantine, compared with the saline and normal groups at 1, 2, 4 and 8 h. The gene numbers of the 48 sets of DEGs are presented in Table I. The overlapping genes were respectively identified in the three drug groups compared with both the saline and the normal group at different time points, and the gene numbers are presented in Table II. Furthermore, the overlapping genes were screened out at the four time points, and are presented in Table III. *Plin4* was the only overlapping gene among the aforementioned 48 sets of DEGs, and *Plin4, Sgk1, Klf2* and *Dcaf12l1* were overlapping in the striatum samples treated with the three drugs at the different time points (Table III). Furthermore, *Plin4, Sgk1* and *Klf2* were upregulated in the striatum samples treated with the three drugs compared with saline or no treatment, and *Dcaf12l1* was downregulated.

**Figure 1.**
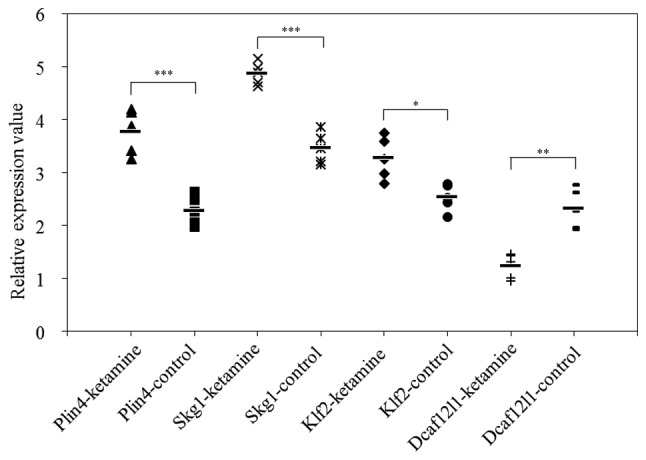
Relative expression levels of *Plin4, Skg1, Klf2* and *Dcaf12l1* in the striatum samples. ***P<0.001, **P<0.01 and *P<0.05 as indicated. -ketamine, ketamine-treated striatum samples; -control, saline-treated striatum samples; SGK1, serum/glucocorticoid regulated kinase 1; Plin4, perilipin 4; Klf2, Kruppel like factor 2; Dcaf12l1, DDB1 and CUL4 associated factor 12 like 1.

**Figure 2.**
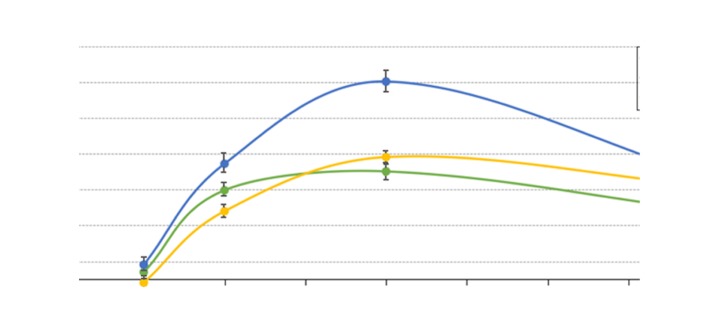
Expression of *Plin4* in the hippocampus samples treated with KET, PHE and MEM. KET, ketamine; PHE, phencyclidine; MEM, memantine; Plin4, perilipin 4.

**Figure 3.**
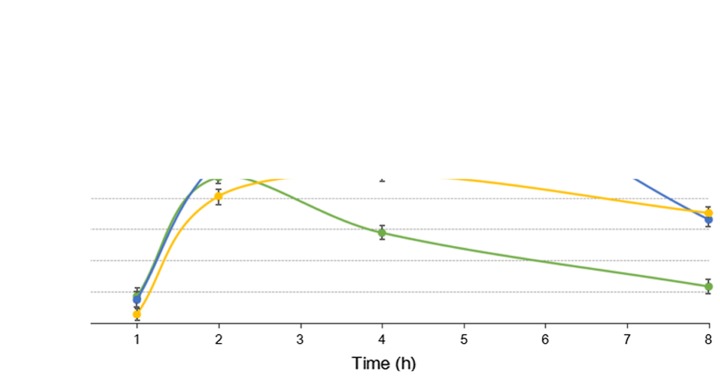
Expression of *Plin4* in the striatum samples treated with KET, PHE and MEM. KET, ketamine; PHE, phencyclidine; MEM, memantine; Plin4, perilipin 4.

The results of RT-qPCR are presented in Fig. 1. The mRNA expression levels of *Plin4, Sgk1* and *Klf2* were significantly higher in the striatum samples of the ketamine group compared with the control group (P<0.05), while the mRNA expression of *Dcaf12l1* was significantly lower compared with the control group (P<0.05). In addition, the expression of *Plin4* in the hippocampus and striatum following treatment with the three drugs is presented in Figs. 2 and 3, respectively. The results indicated that the expression of *Plin4* was highest both in the hippocampus and striatum after treatment with ketamine compared with phencyclidine and memantine.

#### GO terms and pathway analysis

The enriched GO terms and KEGG pathways of *Plin4, Sgk1, Klf2* and *Dcaf12l1* were selected, and the results are presented in Tables IV and V. *Plin4* was enriched in 1 KEGG pathway; *Sgk1* was enriched in 5 GO terms and 4 KEGG pathways; and *Klf2* was enriched in 4 GO terms and 3 KEGG pathways. Among these, *Sgk1* and *Klf2* were enriched in the ‘forkhead box O (FoxO) signaling pathway’, and *Sgk1* was additionally enriched in the ‘mechanistic target of rapamycin kinase (mTOR) signaling pathway’. *Plin4* and *Dcaf12l1* were enriched in no GO terms, and *Dcaf12l1* was enriched in no pathways.

#### PPI network

**Figure 4.**
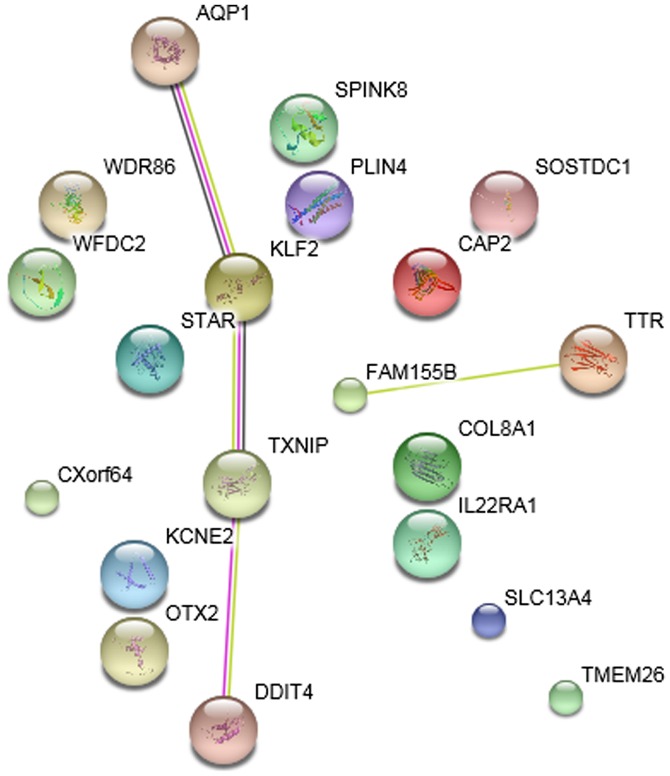
Protein-protein interaction network of differentially expressed genes in the striatum samples treated with ketamine. Small nodes represent proteins of unknown 3D structure, large nodes represent proteins with known or predicted 3D structure. Colored nodes represent query proteins and the first shell of interactors. Interactors with purple edges represent known experimentally determined interactions, brilliant green edges represent textmining interactions and dark edges represent co-expression interactions. STRING, search tool for the retrieval of interacting genes/proteins.

**Figure 5.**
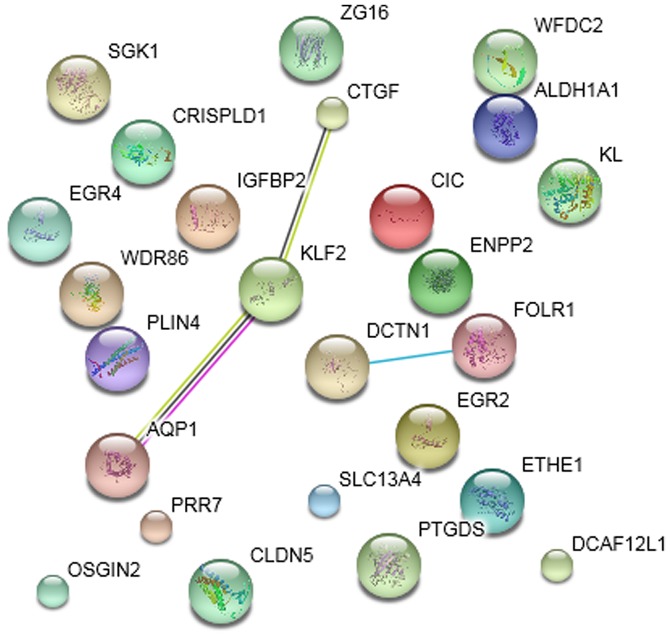
Protein-protein interaction network of differentially expressed genes in the striatum samples treated with phencyclidine. Small nodes represent proteins of unknown 3D structure, large nodes represent proteins with known or predicted 3D structure; colored nodes represent query proteins and first shell of interactors; interactors with light blue lines represent known interactions from STRING databases, purple edges represent known experimentally determined interactions, brilliant green edges represent textmining interactions, dark edges represent co-expression interactions. STRING, search tool for the retrieval of interacting genes/proteins.

**Figure 6.**
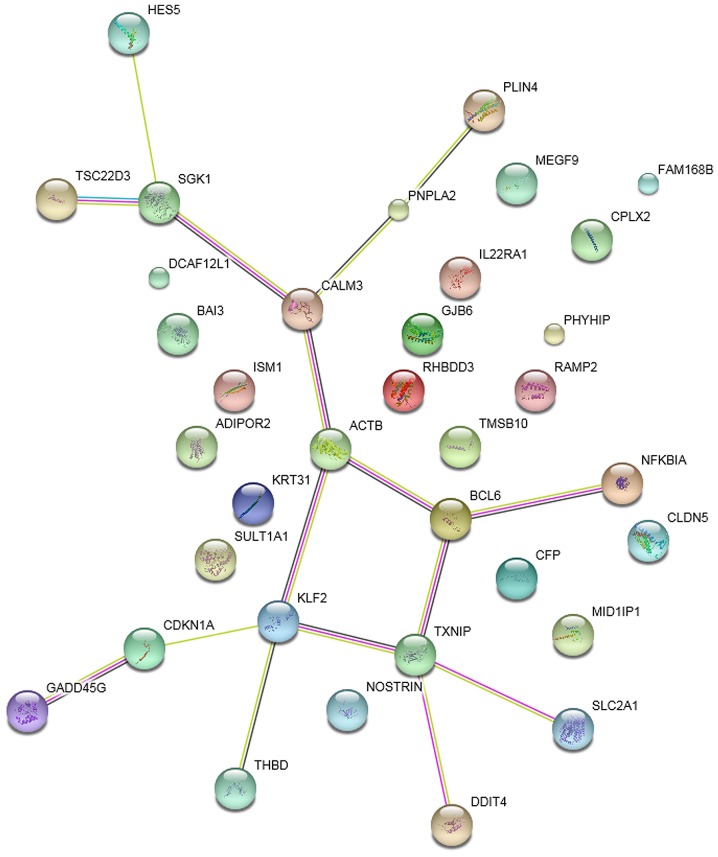
Protein-protein interaction network of differentially expressed genes in the striatum samples treated with memantine. Small nodes represent proteins of unknown 3D structure, large nodes represent proteins with known or predicted 3D structure; colored nodes represent query proteins and first shell of interactors; interactors with light blue lines represent known interactions from STRING databases, purple edges represent known experimentally determined interactions, brilliant green edges represent textmining interactions, dark edges represent co-expression interactions. STRING, search tool for the retrieval of interacting genes/proteins.

The PPI networks of DEGs in the striatum samples treated with ketamine, phencyclidine and memantine compared with normal samples are presented in Figs. 4–6, respectively. The results indicated that *Klf2* was involved in more PPI pairs in these three PPI networks and was therefore the hub gene.

## Discussion

Genetic factors may promote or even cause the occurrence of depression (21). A systematic review found that major histocompatibility complex, class I-related gene polymorphisms, and glutamate decarboxylase *(GAD)* genes (*GAD1* and *GAD2*) contributed to the development of depression (22). One study reported that 5-hydroxytryptamine receptor 2A functional rs6311 polymorphism may modulate the severity of depression symptoms in children with autism spectrum disorder (23). Another study reported that glutamate ionotropic receptor kainate type subunit 4 variants were involved in treatment-resistant depression (24). Apoptosis regulator BCL2 was considered to serve a role in mediating the outcome of antidepressant treatment (24). In the current study, rat models of depression were selected to investigate the underlying mechanism of treatment with ketamine. The present study was primarily based on bioinformatics and a comparison between the ketamine and control groups *in vivo* further demonstrated the effect of ketamine during depression, and provided an additional method for exploring the mechanism of action of ketamine. In addition, the underlying mechanism requires further verification in animal models and human trials, however the current results provide a preliminary basis for these.

In the present study, *Plin4, Sgk1, Klf2* and *Dcaf12l1* were the overlapping DEGs in the striatum samples treated with three NMDA receptor antagonists at different time points. Furthermore, the expression levels of *Plin4, Sgk1* and *Klf2* increased in the striatum samples of rat models of depression following administration of ketamine, and the expression of *Dcaf12l1* decreased compared with the control group. Plin4 protein coats lipid droplets in adipocytes to protect them from lipases (25,26). *Plin4* is associated with insulin resistance and obesity risk (27,28). In the current study, *Plin4* was identified as the only overlapping DEG (upregulated) in hippocampus or striatum samples individually treated with ketamine, phencyclidine or memantine compared with the saline or normal groups at 1, 2, 4 and 8 h. SGK1 protein contributes to the regulation of discrete developmental stages and pathological conditions including hypertension, diabetic neuropathy, ischemia, trauma and neurodegenerative diseases (29). Anacker *et al* (30) identified SGK1 as a mediator of the effects of cortisol on neurogenesis and glucocorticoid receptor function, with particular relevance to stress and depression. KLF2 protein is implicated in lung development, embryonic erythropoiesis, epithelial integrity, T-cell viability and adipogenesis (31). Another study by Miller *et al* (32) was performed using ribosome-bound mRNA footprinting and deep sequencing, and confirmed that initiation of protein synthesis is a defining feature of antidepressant dose ketamine in mice; with the use of GO analysis, vasoactive intestinal peptide receptor 2 gene was identified as a potential target for antidepressant action. In the current study, Klf2 was involved in more pairs in the PPI network of DEGs in the striatum samples treated with ketamine, phencyclidine or memantine compared with normal samples. That was to say, *Klf2* was differentially expressed between the groups compared with the control, and that *Klf2* exhibited the highest degree among all proteins in the PPI networks. However, to the best of the authors' knowledge, the association between *Klf2* and depression or NMDA receptor antagonists has not been previously reported. Dcaf12l1 protein is associated with embryonic development and idiopathic nonobstructive azoospermia (33,34). The current study indicated that *Plin4, Sgk1, Klf2* and *Dcaf12l1* were differentially expressed in depression models treated with ketamine, phencyclidine and memantine, which suggested that these genes may be the targets of the NMDA receptor antagonist treatment.

Duman *et al* (35) investigated the signaling pathway underlying the rapid antidepressant effects of ketamine, and the results demonstrated that the effects were associated with the stimulation of mTOR and increased expression levels of synaptic proteins. Li *et al* (36) observed that ketamine rapidly activated the mTOR pathway, leading to increased expression levels of synaptic signaling proteins and increased number and function of new spine synapses in the prefrontal cortex of rats, while inhibition of mTOR signaling completely blocked ketamine-mediated induction of synaptogenesis and behavioral responses in models of depression. The above results indicated that the effects of ketamine are opposite to the synaptic deficits that result from exposure to stress and could contribute to the rapid antidepressant effects of ketamine. The current study also found that *Sgk1*, one of the key DEGs underlying the rapid antidepressant effects of ketamine (37), was enriched in the ‘mTOR signaling pathway’. In addition, the present results indicated that both *Sgk1* and *Klf2* were enriched in the ‘FoxO signaling pathway’. FoxO is a subfamily of the fork head transcription factor family, serving roles in cell fate decisions (38). Polter *et al* (39) observed that FoxO may be a transcriptional target for treatment of anxiety and mood disorders, and serves a potential role in regulating mood-associated behavior. This study further indicated that mice displayed reduced anxiety when FoxO1 was knocked down in the brain (39). Hence, FoxO3a-deficient mice presented with an antidepressant-like behavior (39). The FoxO signaling pathway is considered to be a therapeutic target in cancer (40), and mediates stress responses (41,42). Therefore, it is hypothesized that the mTOR and FoxO signaling pathways may be involved in the underlying mechanism of antidepressant effects of ketamine.

In conclusion, the present study suggested that the mTOR and FoxO signaling pathways may serve roles in the underlying mechanism of antidepressant effects of ketamine, and *Plin4, Sgk1, Klf2* and *Dcaf12l1* may be potential biomarkers for depression and targets for NMDA receptor antagonist treatment of depression.

## Figures and Tables

**Table I.** Gene numbers of the 48 sets of DEGs in hippocampus or striatum samples individually treated with ketamine, phencyclidine or memantine compared with the saline or normal group at 1, 2, 4 and 8 h, respectively.

		Hippocampus				Striatum			
			
Drug	Control	1 h	2 h	4 h	8 h	1 h	2 h	4 h	8 h
Ketamine	Saline	13 (2,11)	7 (6,1)	2 (2,0)	0 (0,0)	113 (23,90)	122 (30,92)	235 (121,114)	103 (41,62)
	Normal	7 (6,1)	12 (5,7)	8 (8,0)	2 (1,1)	50 (36,14)	91 (47,44)	64 (47,17)	26 (23,3)
Phencyclidine	Saline	10 (3,7)	4 (4,0)	4 (1,3)	0 (0,0)	87 (46,41)	106 (18,88)	243 (119,124)	98 (44,53)
	Normal	8 (4,4)	14 (3,11)	15 (10,5)	5 (2,3)	50 (41,9)	43 (9,34)	38 (21,17)	31 (27,4)
Memantine	Saline	4 (4,0)	18 (16,2)	11 (6,5)	1 (0,1)	104 (13,91)	99 (31,68)	520 (241,279)	89 (44,45)
	Normal	7 (7,0)	16 (16,0)	7 (7,0)	1 (1,0)	19 (14,5)	43 (30,13)	134 (60,74)	13 (11,2)

Data are presented as the total number of DEGs (upregulated DEGs, downregulated DEGs). DEGs, differentially expressed genes.

**Table II.** Gene numbers of the overlapping genes in three drug groups compared with both the saline and normal groups.

	Hippocampus				Striatum			
		
Drug	1 h	2 h	4 h	8 h	1 h	2 h	4 h	8 h
Ketamine	3 (2,1)	1 (0,1)	2 (2,0)	0 (0,0)	10 (7,3)	26 (19,7)	7 (5,2)	6 (6,0)
Phencyclidine	4 (2,2)	0 (0,0)	4 (1,3)	0 (0,0)	20 (18,2)	11 (3,8)	2 (1,1)	9 (7,2)
Memantine	4 (4,0)	9 (9,0)	5 (5,0)	0 (0,0)	5 (4,1)	14 (10,4)	40 (18,22)	2 (1,1)

**Table III.** Overlapping genes at the four time points.

Tissue	Drug	Upregulated	Downregulated
Hippocampus	Ketamine Phencyclidine	*Plin4, Sgk1, Gh, Txnip Klf2, Plin4, mtDNA_ND6*	*Ttr mtDNA_ND4L, Stk32c, Hebp1, mt-Nd4l, Prr7*
	Memantine	*Plin4, Klf2, Gadd45g, Mfsd2a, Ddit4, Cdkn1a, LOC240672, Dusp1, Sult1a1, Sgk1, Fos, Egr4, Txnip, Tsc22d3*	
Striatum	Ketamine	*Plin4, Sgk1, Klf2, Il22ra1, Vmn1r78, Slc13a4*, D7Zem2, Txnip, Ddit4, Olfr938, Kcne2, Star, 1500015O10Rik, Tmem26, Wfdc2, Spink8, *Calml4, Olfr247, LOC380910, Ttr, Sostdc1, Cap2, Gm4758, Cox8b, Vmn1r89, Lbp, Col8a1, 932418N15Rik, Tmem28, Prr32, LOC672705, Folr1, Otx2, Aqp1, Clic6, Wdr86*	*Dcaf12l1, Meox1, Olfr837, Slc6a13, Crh, LOC100046930, Fut1, Krt31, Fam171a1, Olfr938, Olfr1168, Fam214b*
	Phencyclidine	*Plin4, Sgk1, Klf2, Kl, Enpp2, Olfr474, Dctn1, A030006E20Rik, D7Zem2, Aqp1, Fgf15, Cox8b*, Wfdc2, Zg16, Folr1, LOC381105, Hbb-b2, Wdr86, Slc13a4, Ptgds, Crispld1, Osgin2, LOC383036, *Igfbp2, Ces1a, Ctgf*	*Dcaf12l1, Aldh1a1, Fbxw15, Egr2, Cyp2c55, Egr4, Cldn5, Prr7, A230065H16Rik, LOC194360, Cic, Ethe1, Olfr1317*
	Memantine	*Plin4, Sgk1, Klf2, Nfkbia, E130102H24Rik*, Cyp2c39, Pnpla2, Slc2a1, Gjb6, Gadd45g, Il22ra1, Megf9, Cdkn1a, Nostrin, BAI3, Ism1, Cfp, Ramp2, Txnip, Bcl6, Sult1a1, Tsc22d3, LOC100044968, 9930108O06Rik, *Adipor2, Thbd, Ddit4*	*Dcaf12l1, LOC331511, Cyp2c55, Phyhip, LOC386233, Mid1ip1, Rpl27a-ps1, LOC381105, Cplx2, Calm3, Fam168b, Actb, Krt31, LOC241621, Pimreg*,
		Megf9, Cdkn1a, Nostrin, BAI3, Ism1, Cfp, Ramp2, Txnip, Bcl6, Sult1a1, Tsc22d3, LOC100044968, 9930108O06Rik, Adipor2, Thbd, Ddit4	*C330049H01Rik, LOC433476, Cldn5, LOC385068, Olfr1362, Hes5, Tmsb10, Rhbdd3, BC037112, LOC382092, LOC382237, LOC673501, LOC381132*

**Table IV.** mRNA levels of *Plin4, Sgk1, Klf2* and *Dcaf12l1* in the striatum samples.

	Relative expression value			
	
Group	*Plin4*	Sgk1	Klf2	*Dcaf12l1*
Ketamine group	3.77±0.38	4.87±0.19	3.27±0.36	1.23±0.21
Control group	2.28±0.25	3.46±0.27	2.53±0.23	2.31±0.34
P-value	<0.0001	<0.0001	0.0402	0.0018
T-value	8.76	12.81	−3.68	7.87

**Table V.** The enriched GO terms of *Sgk1* and *Klf2*.

Gene	GO term	Function
*Sgk1*	GO:0004672	‘Protein kinase activity’
	GO:0004674	‘Protein serine/threonine kinase activity’
	GO:0005524	‘ATP binding’
	GO:0016301	‘Kinase activity′’
	GO:0016740	‘Transferase activity’
*Klf2*	GO:0003677	‘DNA binding’
	GO:0003700	‘Transcription factor activity, sequence-specific DNA binding’
	GO:0008270	‘Zinc ion binding’
	GO:0046872	‘Metal ion binding’

GO, gene ontology; SGK1, serum/glucocorticoid regulated kinase 1; Klf2, Kruppel like factor 2.

**Table VI.** Enriched KEGG pathways of *Plin4, Sgk1* and *Klf2*.

Gene	KEGG pathway	Name
*Sgk1*	mmu04068	‘FoxO signaling pathway’
	mmu04150	‘mTOR signaling pathway’
	mmu04151	‘PI3K-Akt signaling pathway’
	mmu04960	‘Aldosterone-regulated sodium reabsorption’
*Plin4*	mmu03320	‘PPAR signaling pathway’
*Klf2*	mmu04068	‘FoxO signaling pathway’
	mmu04371	‘Apelin signaling pathway’
	mmu05418	‘Fluid shear stress and atherosclerosis’

KEGG, Kyoto Encyclopedia of Genes and Genomes; SGK1, serum/glucocorticoid regulated kinase 1; Plin4, perilipin 4; Klf2, Kruppel like factor 2.

## Data Availability

All data generated or analyzed during the present study are included in this published article.
